# Association of a single nucleotide polymorphism in *SOD2* with susceptibility for the development of diabetic nephropathy in patients with type 2 diabetes: A Saudi population study

**DOI:** 10.1002/edm2.449

**Published:** 2023-09-12

**Authors:** Samar Sultan, Meshari Alharbi, Nuha Alrayes, Nehad Makki, Hanan Faruqui, Lama Basuni, Amani Alhozali, Reham Abdulnoor, Anwar Borai, Abdullah Almalki, Abdullah Alzahrani, Reem Alamoudi, Mazin Almaghrabi

**Affiliations:** ^1^ Medical Laboratory Sciences, Faculty of Applied Medical Sciences King Abdulaziz University Jeddah Saudi Arabia; ^2^ King Abdulaziz Medical City National Guard Hospital Jeddah Saudi Arabia; ^3^ Princes Al‐Jawhara center of excellence in research of hereditary disorders, King Abdulaziz University Jeddah Saudi Arabia; ^4^ Department of Medicine, Faculty of Medicine King Abdulaziz University Jeddah Saudi Arabia; ^5^ King Abdullah International Medical Research Center (KAIMRC) King Saud bin Abdulaziz University for Health Sciences (KSAU‐HS), King Abdulaziz Medical City, Ministry of National Guard Jeddah Saudi Arabia; ^6^ King saud bin Abdulaziz university for health sciences, king abdulaziz medical city king Abdullah international research center (KAIMRC) Jeddah Saudi Arabia; ^7^ King Abdulaziz Medical city, College of Medicine King Saud Bin Abdulaziz University for Health Sciences, King Abdullah International Medical Research center Jeddah Saudi Arabia; ^8^ Department of Internal Medicine/Endocrinology King Abdulaziz Medical City Jeddah Saudi Arabia

**Keywords:** antioxidant enzymes, nephropathy, single nucleotide polymorphisms, type 2 diabetes

## Abstract

**Introduction:**

One of the complications of diabetes mellitus (DM) is diabetic nephropathy (DN), which plays a significant role in the progression of end‐stage renal disease. Oxidative stress is implicated in DN pathogenesis, and genetic variations in antioxidant enzymes such as superoxide dismutase 2 (SOD2) and catalase (CAT) may contribute to the susceptibility. This study aimed to investigate the potential association between single nucleotide polymorphisms (SNPs) in antioxidant enzymes, specifically *SOD2* rs4880 and *CAT rs769217*, and the risk of T2D and susceptibility to DN within the Saudi population.

**Methods:**

This case–control study included 150 participants, comprising 50 patients with T2D without DN (group 1), 50 patients with T2D with DN (group 2), and 50 healthy participants (group 3). The samples were genotyped using real‐time PCR for *SOD2* rs4880 and *CAT* rs769217 SNPs. Sanger sequencing was used for validation. Statistical analyses were performed to explore associations between these SNPs and T2D with or without DN.

**Results:**

No significant difference was observed in *CAT* rs769217 expression between the groups. However, a significant difference was observed in *SOD2* rs4880 expression between the healthy controls and patients with T2D with DN (*p* = .028). Furthermore, *SOD2* rs4880 was associated with approximately threefold increased risk of DN in patients with T2D compared to that in healthy participants (odds ratio [OR] = 2.99 [1.31–6.83]). Validation through Sanger sequencing further confirmed these findings.

**Conclusions:**

The findings of this study provide evidence that *SOD2* rs4880 SNP may contribute to inadequate defence by the antioxidant enzyme, SOD2, against DM‐induced oxidative stress and thus cause DN in Saudi patients with T2D. Therefore, *SOD2* rs4880 may serve as a predictive marker to prevent the development and progression of DN in patients with T2D.

## Graphical abstract

Our findings revealed a statistically significant difference between the healthy control group and T2D patients with DN regarding the expression of SOD2 SNP. No significant difference was observed in the expression of CAT SNP between the groups. These data provide evidence that the SOD2 SNP might contribute to improper defence of the SOD2 enzyme against diabetes‐induced oxidative stress and cause the DN in Saudi patients with T2D.

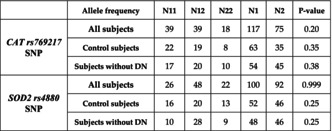

## 1 INTRODUCTION

Type 2 diabetes (T2D) is a complex disorder associated with high blood glucose levels due to low insulin secretion or inadequate insulin action. In the Kingdom of Saudi Arabia, managing diabetes mellitus (DM) has emerged as a significant challenge, with >50% of the population affected by DM or pre‐DM.^1^ Between 1992 and 2010, the incidence rate of T2D increased 2.7 times, as reported by the Ministry of Health. Patients with T2D are predisposed to various other chronic diseases and complications, including diabetic nephropathy (DN), neuropathy, retinopathy and cardiovascular disease.^2^ DN is a common T2D complication that increases morbidity and mortality rates and plays a significant role in the progression of end‐stage renal disease. As previously reported, one in five patients with DM develops end‐stage renal disease in the Saudi population.^3^ Conversely, other studies have documented that T2D accounted for 42.5% of end‐stage renal disease cases in Saudi Arabia in 2011.^4^

The progression of DN involves numerous factors that serve as significant markers. These include a reduction in the glomerular filtration rate (GFR) of <60 mL/min/1.73 m^2^, proteinuria, poorly controlled blood pressure (>130 mm Hg), high creatinine levels (>130 μmol/L) and retinopathy.^3^ Recently, diabetic kidney disease had been linked to oxidative stress, which is caused by an imbalance between free radicals and antioxidants. Oxidative stress is accompanied by the overproduction or improper elimination of molecules such as reactive oxygen species (ROS) and reactive nitrogen species. The oxidation–reduction reaction, facilitated by enzymes such as nicotinamide adenine dinucleotide phosphate (NADPH) and xanthine oxidase, results in the production of superoxide when an oxygen molecule gains an electron.^5^ Four pathways implicated in diabetic complications have been identified: protein kinase C, polyol, hexosamine and advanced glycation end products.^6^

Oxidative stress is involved in inflammation,^7^ a significant characteristic of DN.^8^ The Care Time study showed increased inflammation in patients with DN.^9^ Additionally, various inflammatory markers and cytokines such as mean platelet volume,^10^ serum uric acid,^11^ monocyte/lymphocyte ratio in hemogram,^12^ and neuregulin^13^ have been implicated in DN.

*Manganese superoxide dismutase* (MN‐SOD), also known as superoxide dismutase 2 (SOD2), is an important enzyme that eliminates ROS generated during oxidative phosphorylation reactions in the mitochondrial respiratory chain—a significant source of intracellular ROS. Polymorphisms of the *SOD2* gene have been identified at amino acid 16 (Val16Ala or rs4880), which may interrupt its ability to eliminates ROS.^14^ Catalase (CAT) is a significant enzyme that acts as an antioxidant by degrading hydrogen peroxide to water and oxygen. Mutations in the CAT enzyme can cause functional defects, leading to neurological disorders such as Alzheimer's disease and Parkinson's disease; metabolic disorders such as DM and hypertension; and cancer.^15^ Other gene polymorphisms have also been recognized as predisposing risk factors and therapeutic targets for DM with or without kidney diseases. These include sirtuin 1 (*SIRT1*) rs12778366 and rs3758391,^16^ β1‐Adrenegic receptor (*ADRB‐1*) rs1801253C/G,^17^ *NR1H2* gene encoding *LXRβ* (28,514,894 and rs2303044), ^18^ *miR143/145* cluster,^19^ and *miR29a* (rs157907A/G).^20^

Insufficient cellular antioxidative defence caused by single nucleotide polymorphisms (SNPs) in these antioxidant enzymes may be involved in DN‐associated T2D processes. Thus, this study aimed to investigate the potential association between the *SOD2* rs4880 and *CAT* rs769217 SNPs and T2D with or without DN.

## 2 METHODS

### 2.1 Participants

In this cross‐sectional case–control study, blood samples were collected from 150 participants. The participants were divided into three groups: those with T2D but without DN (group 1; *n* = 50), those with T2D with DN (group 2; *n* = 50), and healthy participants without diabetes (group 3 [control]; *n* = 50). These participants sought care at the Diabetes Center in the National Guard Hospital, Jeddah, Saudi Arabia between July 2021 and December 2021. Written informed consent was obtained from all participants after a thorough explanation of the purpose of the study. Additionally, participants were assured that all personal information would be kept confidential. The study was approved by the institutional review board at the National Guard Hospital (IRB#SP21J/419/09).

The control group comprised healthy participants with no history of DM, kidney disease, hypertension, hepatic disease, or other relevant conditions. For group 1, those with a haemoglobin A1c (HbA1c) level ≥6.5%, fasting blood glucose ≥126 mg/dL, 2‐h oral glucose tolerance test blood glucose level ≥ 200 mg/dL, and random blood glucose ≥200 mg/dL were classified as having DM, in accordance with the American Diabetes Association criteria from 2011.^21^ In group 2, participants with an estimated GFR (eGFR) <60 mL/min/1.73 m^2^ and a urine albumin/creatinine ratio > 30 mg/g were classified as having DM with DN, following the American Diabetes Association criteria.^21^ The biochemical parameters of the donors were obtained from their medical records.

### 2.2 Genotype analysis

Whole blood samples (3 mL) were collected from each participant in labelled ethylenediaminetetraacetic acid (EDTA) tubes. Genomic DNA was isolated using the DNeasy kit (Qiagen) as per the manufacturer's instructions and stored at −20°C until further processing. Genotyping of *SOD2* rs4880 and *CAT* rs769217 antioxidant enzyme polymorphisms was conducted using the TaqMan assay and real‐time PCR (Applied Biosystem), following the manufacturer's protocol. Briefly, 20 μL of the mixture was added to each well of a 96‐well plate. The mixture included 10 μL of 2× Taq Man Genotyping Master Mix, 1 μL of 20× SNP Genotyping assay, 2 μL of DNA, and 7 μL of H_2_O. The genotyping reference SNP IDs for detection of *SOD2* and *CAT* are rs4880(assay ID:C_8709053_10) and rs769217(assay C_3102907_10), respectively. Subsequently, the microtiter plate was placed in a thermal cycler for enzyme activation at 95°C for 10 min, followed by 40 cycles of denaturation at 95°C for 15 s, annealing at 60°C for 1 min, and extension at 60°C for 1 min, according to the manufacturer's recommendation.

For validation, Sanger sequencing was used. A mixture of 20 μL was prepared for conventional PCR. The mixture contained 10 μL of the master mix, 1 μL of forward primer and 1 μL of reverse primer. The forward and reverse primers used for *SOD2* rs4880 were GGTAGCACCAGCACTAGCAG and TCAGCCTGGAACCTACCCTT, respectively. For *CAT* rs769217, the forward and reverse primers were AAGTAGCGGGAAAGGCAGAA and CACCTGGGGAGCACCTTTAC, respectively. Subsequently, 6 μL of distilled water (dH_2_O) was added, followed by 2 μL of DNA. A 2% agarose gel was used for electrophoresis, and the labelled gene was detected using a gel imaging system (Azure Biosystem). Exosap (2 μL) was added to 5 μL of the DNA PCR product and incubated at 37°C for 30 min, followed by 80°C for 15 min. The sequencing reaction was performed using 4 μL of Big‐Dye, 0.4 μL of primers, 4.6 μL of dH_2_O, and 1 μL of DNA in a total volume of 10 μL. The thermocycler settings were as follows: initial denaturation at 96°C for 1 min (one cycle), followed by 25 cycles of denaturation, annealing, and extension at 96°C for 10 s, 46°C for 5 s, and 60°C for 4 min, respectively. The reaction was then held at 4°C for one cycle. SAM solution (45 μL) (Thermo‐Scientific) and 10 μL of beads (Thermo‐Scientific) were added to the mixture, followed by vortexing for 45 min at 3000 rpm. The mixture was centrifuged for 2 min at 1000 rpm, and 20 μL of the supernatant was transferred to a 96‐well plate, which was sealed and loaded in a Sanger sequencing analyser (Thermo‐Scientific). The Snap Gene program was used to analyse the data.

### 2.3 Statistical analysis

The Statistical Package for Social Sciences (IBM, SPSS, version 28) was used to describe and compare the demographic, clinical, and laboratory findings among the three groups. Qualitative variables were expressed as frequencies and percentages, while numerical continuous variables were expressed as mean ± standard deviation (SD). The Kolmogorov–Smirnov and Shapiro–Wilk tests were applied to explore the normality of quantitative variables. Accordingly, parametric or non‐parametric statistical tests of significance were applied. A statistical power of 0.8 was chosen, indicating a 80% probability to correctly reject the null hypothesis. The effect size (*d*) was calculated by dividing the estimated difference between two compared groups by their pooled estimated SD. The minimum sample size for the study groups was determined using the Raosoft Online sample size calculator.^22^ This calculation resulted in a minimum requirement of 45 participants per study group, factoring in a 5% margin of error, 95% confidence level, and a reported 3% incidence of DN among patients with T2D.^23^ However, the sample size was increased to 50 participants in each study group to enhance the sample robustness. *χ*2 test was used to compare qualitative variables, while Student's *t*‐test and one‐way analysis of variance (ANOVA) were used to compare the arithmetic means of continuous variables between two or more groups, respectively. Charts and graphs for diagnostic workup and molecular testing were constructed. Statistical significance was set at *p* < .05.

## 3 RESULTS

### 3.1 Clinical characteristics of the participants

A total of 150 participants were enrolled in this study, evenly distributed across three groups: T2D without DN (group 1; *n* = 50), T2D with DN (group 2; *n* = 50) and control (group 3; *n* = 50). Their demographic data are summarized in Table 1.

**TABLE 1:** Clinical characteristic of the study subjects.

Characteristics	Control subjects	T2D subjects without DN	T2D subjects with DN	*p*‐Value
Age	40.1 ± 12.2	60.5 ± 8.1	67.4 ± 6.4	<0.001*
Gender (%)	50	49	50	0.426*
Male	24 (48.0)	27 (55.1)	21 (42.0)	
Female	26 (52.0)	22 (44.9)	29 (58.0)	
Family history of T2D (%)	49	50	50	<0.001*
No	35 (71.4)	24 (48.0)	16 (32.0)	
Yes	14 (28.6)	26 (52.0)	34 (68.0)	
Consanguinity	49	50	50	<0.001*
No	38 (77.6)	11 (22.0)	23 (46.0)	
Yes	11 (22.4)	39 (78.0)	27 (54.0)	
BMI	33.3 ± 14.5	31.1 ± 4.6	32.8 ± 6.8	0.511*
Albumin	42.7 ± 3.6	41.8 ± 4.8	39.3 ± 3.4	<0.001*
AST	17.9 ± 11.1	21.9 ± 15.5	16.8 ± 6.3	0.074*
ALT	22.5 ± 18.7	27.7 ± 19.9	16.2 ± 6.5	0.003*
Creatinine	77.2 ± 16.8	81.7 ± 31.1	193.0 ± 102.1	<0.001*
EGFR	89.6 ± 17.5	84.3 ± 17.5	35.8 ± 17.9	<0.00*
UACR		2.3 ± 2.7	129.4 ± 183.7	<0.001°
Triglyceride	1.3 ± 0.8	1.5 ± 0.6	1.7 ± 0.9	0.119*
Cholesterol	5.0 ± 0.9	4.2 ± 1.0	4.2 ± 0.9	<0.001*
HDL	1.3 ± 0.4	1.0 ± 0.2	1.1 ± 0.2	<0.001*
LDL	3.1 ± 0.8	2.6 ± 0.8	2.4 ± 0.9	<0.001*
FBG	5.2 ± 0.5	8.9 ± 3.6	9.9 ± 3.7	<0.001*
HA1C	5.3 ± 0.4	8.2 ± 1.6	8.1 ± 1.8	<0.001*
SBP	118.9 ± 10.9	137.7 ± 19.8	144.5 ± 18.7	<0.001*
DBP	75.0 ± 0.7	75.4 ± 12.8	67.7 ± 14.4	0.002*

Abbreviations: AST, aspartate aminotransferase; ALT, alanine aminotransferase; BMI, body mass index; DBP, diastolic blood pressure; EGFR, estimated glomerular filtration rate; FBG, fasting blood glucose; HDL, high density lipoprotein; HbA1c, haemoglobin a1c; LDL, low density lipoprotein; SBP, systolic blood pressure; UACR, urine albumin creatinine ratio.

* Statistically significant.

### 3.2 Comparing the expression of *SOD2* rs4880 and *CAT* rs769217 between healthy participants (control group 3) and those with T2D but without DN (group 1)

**FIGURE 1:**
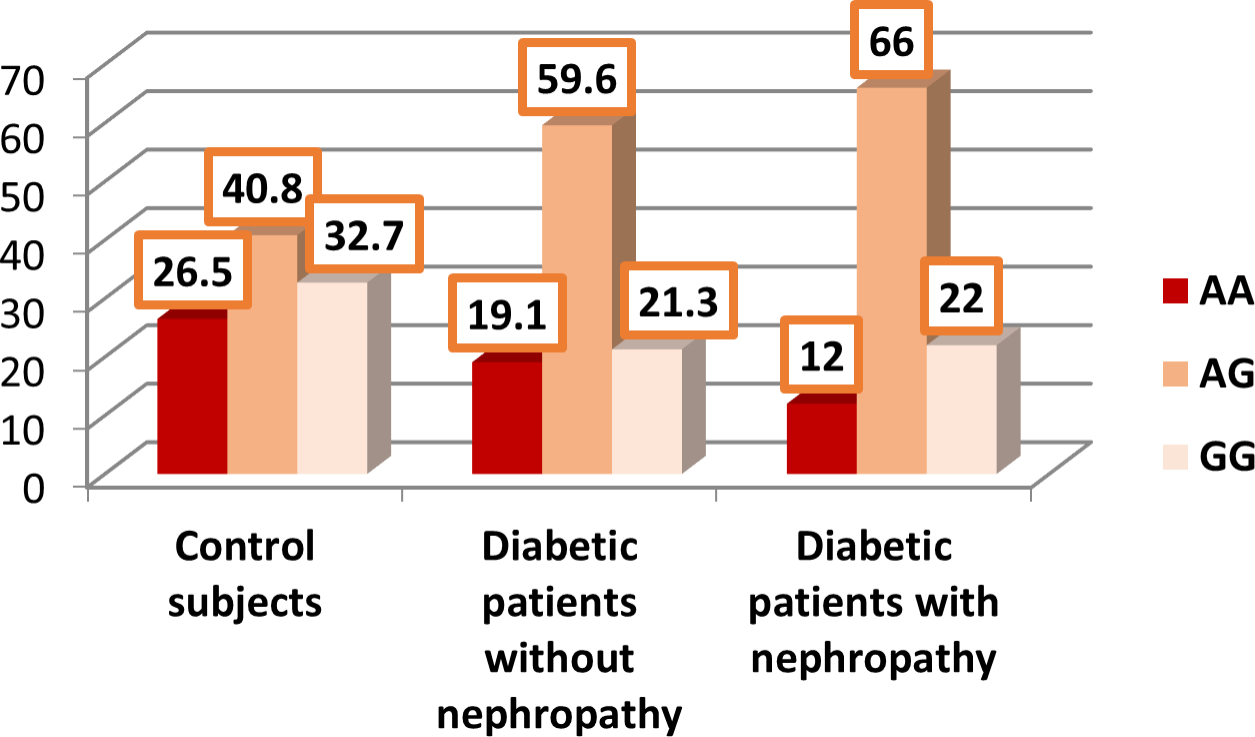
Distribution of the gene encoding the antioxidant enzyme (*SOD2* rs4880) in controls and participants with type 2 diabetes mellitus (T2D) with and without diabetic nephropathy (DN).

**FIGURE 2:**
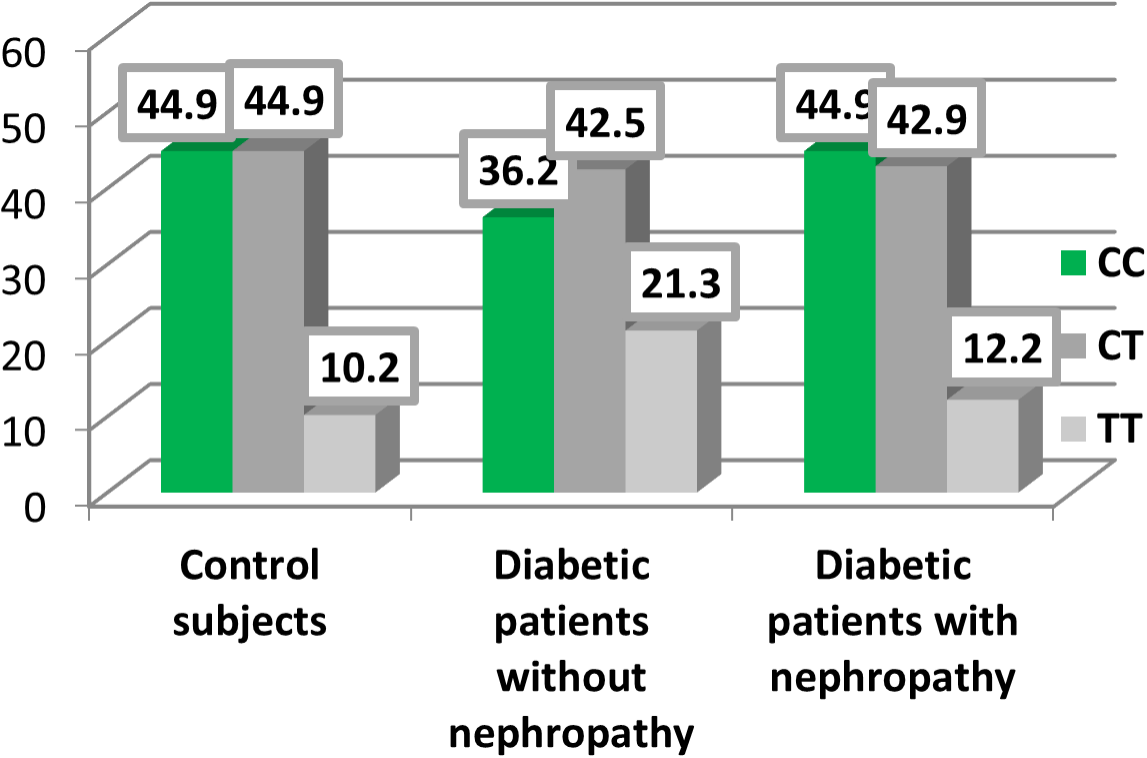
Distribution of the gene encoding the antioxidant enzyme (*CAT* rs769217) in controls and participants with type 2 diabetes mellitus (T2D) with and without diabetic nephropathy (DN).

As presented in Table 2 and Figures 1 and 2, no statistically significant difference was observed in the expression of *SOD2* rs4880 and *CAT* rs769217 between healthy controls (group 3) and participants with T2D but without DN (group 1).

**TABLE 2:** Comparing control subjects and T2D without DN regarding the *CAT rs769217* and *SOD2 rs4880* SNP.

SNP	Allele frequency	Control subjects	Subjects without DN	Genotype frequency	Control subjects *N* = 49	Subjects without DN *N* = 47	χ2	*p*‐Value
*CAT rs769217*	C (117)	63 (64.0)	54 (57.0)	CC	22 (44.9)	17 (36.2)	0.848	0.655
	T (75)	35 (36.0)	40 (43)	CT	19 (38.8)	20 (42.6)		
				TT	8 (16.3)	10 (21.3)		
*SOD2 rs4880*	G (92)	52 (53.0)	48 (51.0)	GG	16 (32.7)	10 (21.3)	3.40	0.182
	A (100)	46 (47)	46 (49)	AG	20 (40.8)	28 (59.6)		
				AA	13 (26.5)	9 (19.1)		

*Note*: Values represent numbers (percentages), *χ*2 = Pearson Chi‐Square score test, *p*‐Value calculated by a Chi‐Square test.

Furthermore, Table 3 presents the Hardy–Weinberg equilibrium (HWE) for the *CAT* rs769217 and *SOD2* rs4880 SNPs between participants in groups 1 and 3. The HWE is a crucial concept in population genetics, describing the expected allele and genotype distribution under certain conditions. It assumes factors such as no selection, mutation, gene flow, random mating and large populations to maintain stable allele frequencies. Deviations from HWE may indicate various genetic phenomena such as selection, migration, or genetic drift.

**TABLE 3:** Exact test for Hardy–Weinberg equilibrium for *CAT rs769217* SNP and *SOD2 rs4880* SNP between control subjects and T2D without DN (*n* = 96).

	Allele frequency	N11	N12	N22	N1	N2	*p*‐Value
*CAT rs769217* SNP	All subjects	39	39	18	117	75	0.20
	Control subjects	22	19	8	63	35	0.35
	Subjects without DN	17	20	10	54	45	0.38
*SOD2 rs4880* SNP	All subjects	26	48	22	100	92	0.999
	Control subjects	16	20	13	52	46	0.25
	Subjects without DN	10	28	9	48	46	0.25

The results suggest that the genotype frequencies in each group do not significantly differ from those expected under HWE. This implies that the allele and genotype frequencies for the rs769217 and rs4880 SNPs are likely in equilibrium, and not subject to strong evolutionary forces or selection in this population within two groups (*p* > .05).

Furthermore, Tables 4 and 5 show the association of *CAT* rs769217 and *SOD2* rs4880 SNPs with the participants in group 1. No association (*p* > .05) was found in any of the examined genetic inheritance models for both SNPs. For *CAT* rs769217 SNP, log‐additive (odds ratio (OR) = 1.29 [0.75–2.22]), over‐dominant (OR = 1.17 [0.52–2.64]), recessive (OR = 1.39 [0.49–3.88]), dominant (OR = 1.44 [0.63–3.26]), and co‐dominant (OR1 = 1.36 [0.56–3.32] and OR2 = 1.62 [0.53–4.98]) models did not show significant association. For *SOD2* rs4880 SNP, log‐additive (OR = 1.08 [0.61–1.91]), over‐dominant (OR = 2.14 [0.95–4.83]), recessive (OR = 0.66 [0.25–1.72]), dominant (OR = 1.79 [0.72–4.50]), and co‐dominant (OR1 = 2.24 [0.84–5.95] and OR2 = 1.11 [0.35–3.54]) models did not show significant association.

**TABLE 4:** Single locus analysis for the association between *CAT rs769217* and T2D in co‐dominant, dominant, recessive, over‐dominant, and log‐additive modes (*n* = 96, crude analysis).

Model	Genotype	Control subjects N = 49	Subjects without DN *N* = 47	OR (95% CI)	*p*‐Value
Codominant	C/C	22 (44.9)	17 (36.2)	1.0	0.655
	C/T	19 (38.8)	20 (42.6)	1.36 (0.56–3.32)	
	T/T	8 (16.3)	10 (21.3)	1.62 (0.53–4.98)	
Dominant	C/C	22 (44.9%)	17 (36.2%)	1.0	0.38
	C/T–T/T	27 (55.1%)	30 (63.8%)	1.44 (0.63–3.26)	
Recessive	C/C‐C/T	41 (83.7%)	37 (78.7%)	1.0	0.53
	T/T	8 (16.3%)	10 (21.3%)	1.39 (0.49–3.88)	
Over dominant	C/C‐T/T	30 (61.2%)	27 (57.5%)	1.0	0.71
	C/T	19 (38.8%)	20 (42.5%)	1.17 (0.52–2.64)	
Log‐additive	–	–	–	1.29 (0.75–2.22)	0.36

**TABLE 5:** Single locus analysis for the association between *SOD2 rs4880* and T2D in co‐dominant, dominant, recessive, over‐dominant, and log‐additive modes (*n* = 96, crude analysis).

Model	Genotype	Control subjects *N* = 49	Subjects without DN *N* = 47	OR (95% CI)	*p‐*Value
Codominant	G/G	16 (32.6%)	10 (21.3%)	1.0	0.18
	A/G	20 (40.8%)	28 (59.6%)	2.24 (0.84–5.95)	
	A/A	13 (26.5%)	9 (19.1%)	1.11 (0.35–3.54)	
Dominant	G/G	16 (32.6%)	10 (21.3%)	1.0	0.21
	A/G‐A/A	33 (67.3%)	37 (78.7%)	1.79 (0.72–4.50)	
Recessive	G/G‐A/G	36 (73.5%)	38 (80.8%)	1.0	0.39
	A/A	13 (26.5%)	9 (19.1%)	0.66 (0.25–1.72)	
Over dominant	G/G‐A/A	29 (59.2%)	19 (40.4%)	1.0	0.065
	A/G	20 (40.8%)	28 (59.6%)	2.14 (0.95–4.83)	
Log‐additive	–	–	–	1.08 (0.61–1.91)	0.78

### 3.3 Comparing the expression of *SOD2* rs4880 and *CAT* rs769217 between control participants (group 3) and those with T2D and DN (group 2)

Tables 6 and Figures 1 and 2 present a comparison of the expression of *SOD2* rs4880 and *CAT* rs769217 between the control group (group 3) and participants with T2D and DN (group 2). A statistically significant difference was observed in the expression of *SOD2* rs4880 between participants in groups 2 and 3. Specifically, 40.8% of participants in the control group expressed *AG* alleles compared to 67.3% in group 2. Additionally, 26.5% and 32.7% of control participants expressed the *AA* and *GG* alleles, respectively, compared to 12.5% and 20.4% of participants in group 2 *(p =* .028). However, no significant difference was observed between the two groups regarding the expression of *CAT* rs769217 (Table 6).

**TABLE 6:** Comparing control subjects and T2D with DN regarding the *CAT rs769217* and *SOD2 rs4880* SNP.

SNP	Allele frequency	Control subjects	Subjects with DN	Genotype frequency	Control subjects *N* = 49	Subjects with DN *N* = 49	χ2	*p*‐Value
*CAT rs769217*	C (128)	63 (64.0)	65 (66.0)	CC	22 (44.9)	22 (44.9)	0.386	0.852
	T (68)	35 (36.0)	33 (34.0)	CT	19 (44.9)	21 (42.9)		
				TT	8 (10.2)	6 (12.2)		
*SOD2 rs4880*	G (105)	52 (53.0)	53 (54.0)	GG	16 (32.7)	10 (20.4)	7.152	0.028*
	A (91)	46 (47.0)	45 (46.0)	AG	20 (40.8)	33 (67.3)		
				AA	13 (26.5)	6 (12.5)		

*Note*: Values represent numbers (percentages), *χ*2 = Pearson Chi‐Square score test, and *p*‐value calculated by a Chi‐Square test.

The HWE for *CAT* rs769217 and *SOD2* rs4880 SNPs between group 1 and 2 is presented in Table 7. There was no detected deviation from the HWE in either group for *CAT* rs769217 SNP (*p* > .05). Additionally, no deviation from the HWE was detected in the control group for *SOD2* rs4880 SNP (*p* > .05). However, a significant deviation from the HWE was observed in group 2 for *SOD2* rs4880 SNP (*p* = .022).

**TABLE 7:** Exact test for Hardy–Weinberg equilibrium for *CAT rs769217* SNP and *SOD2 rs4880* SNP between control subjects and T2D with DN (*n* = 98).

	Allele frequency	N11	N12	N22	N1	N2	*p*‐Value
*CAT rs769217* SNP	All subjects	44	40	14	128	68	0.37
	Control subjects	22	19	8	63	35	0.35
	Subjects with DN	22	21	6	65	33	0.76
*SOD2 rs4880* SNP	All subjects	26	53	19	105	91	0.54
	Control subjects	16	20	13	52	46	0.25
	Subjects with DN	10	33	6	53	45	0.022*

Furthermore, Tables 8 and 9 show the association of *CAT* rs769217 and *SOD2* rs4880 SNPs with T2D with DN (group 2) in the study sample. For *CAT* rs769217 SNP, no association (*p* > .05) was found in any of the examined genetic models of inheritance: log‐additive (OR = 0.92 [0.53–1.62]), over‐dominant (OR = 1.18 [0.53–2.65]), recessive (OR = 0.72 [0.23–2.24]), dominant (OR = 1.0 [0.45–2.22]), and co‐dominant (OR1 = 1.11 [0.47–2.60] and OR2 = 0.75 [0.22–2.52]).

**TABLE 8:** Single locus analysis for the association between *CAT rs769217* and DN in co‐dominant, dominant, recessive, over‐dominant, and log‐additive modes. (*n* = 98, crude analysis).

Model	Genotype	Control subjects	Subjects with DN	OR (95% CI)	*p*‐Value
Codominant	C/C	22 (44.9)	22 (44.9)	1.0	0.82
	C/T	19 (44.9)	21 (42.9)	1.11 (0.47–2.60)	
	T/T	8 (10.2)	6 (12.2)	0.75 (0.22–2.52)	
Dominant	C/C	16 (32.6%)	10 (21.3%)	1.0	0.99
	C/T–T/T	33 (67.3%)	37 (78.7%)	1.00 (0.45–2.22)	
Recessive	C/C‐C/T	36 (73.5%)	38 (80.8%)	1.0	0.56
	T/T	13 (26.5%)	9 (19.1%)	0.72 (0.23–2.24)	
Over dominant	C/C‐T/T	29 (59.2%)	19 (40.4%)	1.0	0.68
	C/T	20 (40.8%)	28 (59.6%)	1.18 (0.53–2.65)	
Log‐additive	–	–	–	0.92 (0.53–1.62)	0.77

*Note*: Values represent numbers (percentages), χ2 = Pearson Chi‐Square score test, *p*‐value ^a^ calculated by a Chi‐Square test, OR = odds ratio, *p*‐value ^b^ calculated by simple logistic regression analysis.

**TABLE 9:** Single locus analysis for the association between *SOD2 rs4880* and DN in co‐dominant, dominant, recessive, over‐dominant, and log‐additive modes. (*n* = 98, crude analysis).

Model	Genotype	Control subjects	Subjects with DN	OR (95% CI)	*p*‐Value
Codominant	G/G	16 (32.6%)	10 (20.4%)	1.0	0.026*
	A/G	20 (40.8%)	33 (67.3%)	2.64 (1.01–6.93)	
	A/A	13 (26.5%)	6 (12.2%)	0.74 (0.21–2.57)	
Dominant	G/G	16 (32.6%)	10 (20.4%)	1.0	0.17
	A/G ‐ A/A	33 (67.3%)	39 (79.6%)	1.89 (0.76–4.73)	
Recessive	G/G ‐ A/G	36 (73.5%)	43 (87.8%)	1.0	0.72
	A/A	13 (26.5%)	6 (12.2%)	0.39 (0.13–1.12)	
Over dominant	G/G ‐ A/A	29 (59.2%)	16 (32.6%)	1.0	0.008**
	A/G	20 (40.8%)	33 (67.3%)	2.99 (1.31–6.83)	
Log‐additive	–	–	–	0.96 (0.53–1.72)	0.88

However, for *SOD2* rs4880 SNP, a statistically significant association was observed with DN risk in the co‐dominant (*p* = .026; OR1 = 2.64 [1.01–6.93] and OR2 = 0.74 [0.21–2.57]), and over‐dominant models (*p* = .008; OR = 2.99 [1.31–6.83]). However, the other models showed no significant association between the *SOD2* rs4880 and DN (*p* > .05): log‐additive (OR = 0.96 [0.53–1.72]), recessive (OR = 0.39 [0.13–1.12]), and dominant (OR = 1.89 [0.76–4.73]).

### 3.4 Comparing the expression of *SOD2* rs4880 and *CAT* rs769217 between participants with T2D but without DN (group 1) and those with DN (group 2)

Table 10 and Figures 1 and 2 present a comparison of the expression of *SOD2* rs4880 and *CAT* rs769217 between groups 1 and 2. No statistically significant difference was observed in the expression of *CAT* rs769217 and *SOD2* rs4880 between the participants in the two groups (*p* > .05). No deviation from the HWE was detected in either group for *CAT* rs769217 (*p* > .05) (Table 11). However, a significant deviation from the HWE was detected in all participants (*p* = .013) and in those with T2D and DN (*p* = .022) for *SOD2* rs4880.

**TABLE 10:** Comparing the expression of *CAT rs769217* and *SOD2 rs4880* SNP between group 1 and group 2.

SNP	Allele frequency	Subjects without DN	Subjects with DN	Genotype frequency	Subjects without DN *N* = 47	Subjects with DN *N* = 49	χ2	*p*‐Value
*CAT* rs769217	C (119)	54 (57.0)	65 (66.0)	CC	17 (36.2)	22 (44.9)	1.624	0.444
	T (73)	40 (43.0)	33 (34.0)	CT	20 (42.2)	21 (42.9)		
				TT	10 (21.3)	6 (12.2)		
*SOD2* rs4880	G (101)	48 (54.0)	53 (54.0)	GG	10 (21.3)	10 (20.9)	0.969	0.616
	A (91)	46 (49.0)	54 (46.0)	AG	28 (59.6)	33 (67.3)		
				AA	9 (19.1)	6 (12.2)		

*Note*: Values represent numbers (percentages), *χ*2 = Pearson Chi‐Square score test, and *p*‐value calculated by a Chi‐Square test.

**TABLE 11:** exact test for Hardy–Weinberg equilibrium for *CAT rs769217* SNP and *SOD2 rs4880* SNP between group 1 and group 2 (*n* = 100).

	Allele frequency	N11	N12	N22	N1	N2	*p*‐Value
*CAT rs769217* SNP	All subjects	39	41	16	119	73	0.39
	Control subjects	17	20	10	54	40	0.38
	Subjects with DN	22	21	6	65	33	0.76
*SOD2 rs4880* SNP	All subjects	20	61	15	101	91	0.013*
	Control subjects	10	28	9	48	46	0.25
	Subjects with DN	10	33	6	53	45	0.022*

Finally, Tables 12 and 13 provide the association analysis of *CAT* rs769217 and *SOD2* rs4880 with DN in the study sample. No significant association was found in the examined genetic inheritance models for both SNPs (*p* > .05). For *CAT rs769217*, log‐additive (OR = 0.71 [0.40–1.24]), over‐dominant (OR = 1.01 [0.45–2.27]), recessive (OR = 0.52 [0.17–1.56]), dominant (OR = 0.70 [0.31–1.58]), and co‐dominant (OR1 = 0.81 [0.34–1.96] and OR2 = 0.46 [0.14–1.53]). For *SOD2* rs4880 SNP, log‐additive (OR = 0.85 [0.43–1.65]), over‐dominant (OR = 1.40 [0.61–3.22]), recessive (OR = 0.59 [0.19–1.81]), dominant (OR = 1.05 [0.39–2.82]), and co‐dominant (OR1 = 1.18 [0.43–3.24] and OR2 = 0.67 [0.17–2.58]).

**TABLE 12:** Single locus analysis for the association between *CAT rs769217* and DN in co‐dominant, dominant, recessive, over‐dominant and log‐additive modes (*n* = 96, crude analysis).

Model	Genotype	Subjects without DN	Subjects with DN	OR (95% CI)	*p*‐Value
Codominant	C/C	17 (36.2%)	22 (44.9%)	1.0	0.44
	C/T	20 (42.5%)	21 (42.9%)	0.81 (0.34–1.96)	
	T/T	10 (21.3%)	6 (12.2%)	0.46 (0.14–1.53)	
Dominant	C/C	17 (36.2%)	22 (44.9%)	1.0	0.38
	C/T–T/T	30 (63.8%)	27 (55.1%)	0.70 (0.31–1.58)	
Recessive	C/C‐C/T	37 (78.7%)	43 (87.8%)	1.0	0.35
	T/T	10 (21.3%)	6 (12.2%)	0.52 (0.17–1.56)	
Over dominant	C/C‐T/T	27 (57.5%)	28 (57.1%)	1.0	0.98
	C/T	20 (42.5%)	21 (42.9%)	1.01 (0.45–2.27)	
Log‐additive	–	–	–	0.71 (0.40–1.24)	0.22

**TABLE 13:** Single locus analysis for the association between *SOD2 rs4880* and DN in co‐dominant, dominant, recessive, over‐dominant, and log‐additive modes (*n* = 96, crude analysis).

Model	Genotype	Subjects without DN	Subjects with DN	OR (95% CI)	*p*‐Value
Codominant	G/G	10 (21.3%)	10 (20.4%)	1.0	0.61
	A/G	28 (59.6%)	33 (67.3%)	1.18 (0.43–3.24)	
	A/A	9 (19.1%)	6 (12.2%)	0.67 (0.17–2.58)	
Dominant	G/G	10 (21.3%)	10 (20.4%)	1.0	0.92
	A/G ‐ A/A	37 (78.7%)	39 (79.6%)	1.05 (0.39–2.82)	
Recessive	G/G–A/G	38 (80.8%)	43 (87.8%)	1.0	0.35
	A/A	9 (19.1%)	6 (12.2%)	0.59 (0.19–1.81)	
Over dominant	G/G ‐ A/A	19 (40.4%)	16 (32.6%)	1.0	0.43
	A/G	28 (59.6%)	33 (67.3%)	1.40 (0.61–3.22)	
Log‐additive	–	–	–	0.85 (0.43–1.65)	0.62

### 3.5 Comparing the expression of *SOD2* rs4880 with fasting blood glucose (FBG), urine albumin creatinine ratio (UACR), and HbA1c

As shown in Table 14, there was no statistically significant difference when comparing the expression of rs4880 with FBG, UACR and HbA1c.

**TABLE 14:** Association between gene encoding antioxidant enzyme rs4880 and levels of FBG, UACR and HbA1c in T2D patients with DN.

Genotype	AA	AG	GG	*p*‐Value*
	*N* = 6	*N* = 33	*N* = 11	
	Mean ± SD	Mean ± SD	Mean ± SD	
FBG	10.5 ± 4.9	9.8 ± 3.7	10.0 ± 3.3	0.913
HbA1c	8.4 ± 2.1	8.2 ± 1.9	7.9 ± 1.2	0.869
UACR	59.9 ± 50.1	143.1 ± 189.8	117.7 ± 204.1	0.689

Abbreviations: FBG, fasting blood glucose; HbA1c, haemoglobin A1c; UACR, urine albumin creatinine ratio.

### 3.6 Validation by Sanger sequencing

**FIGURE 3:**
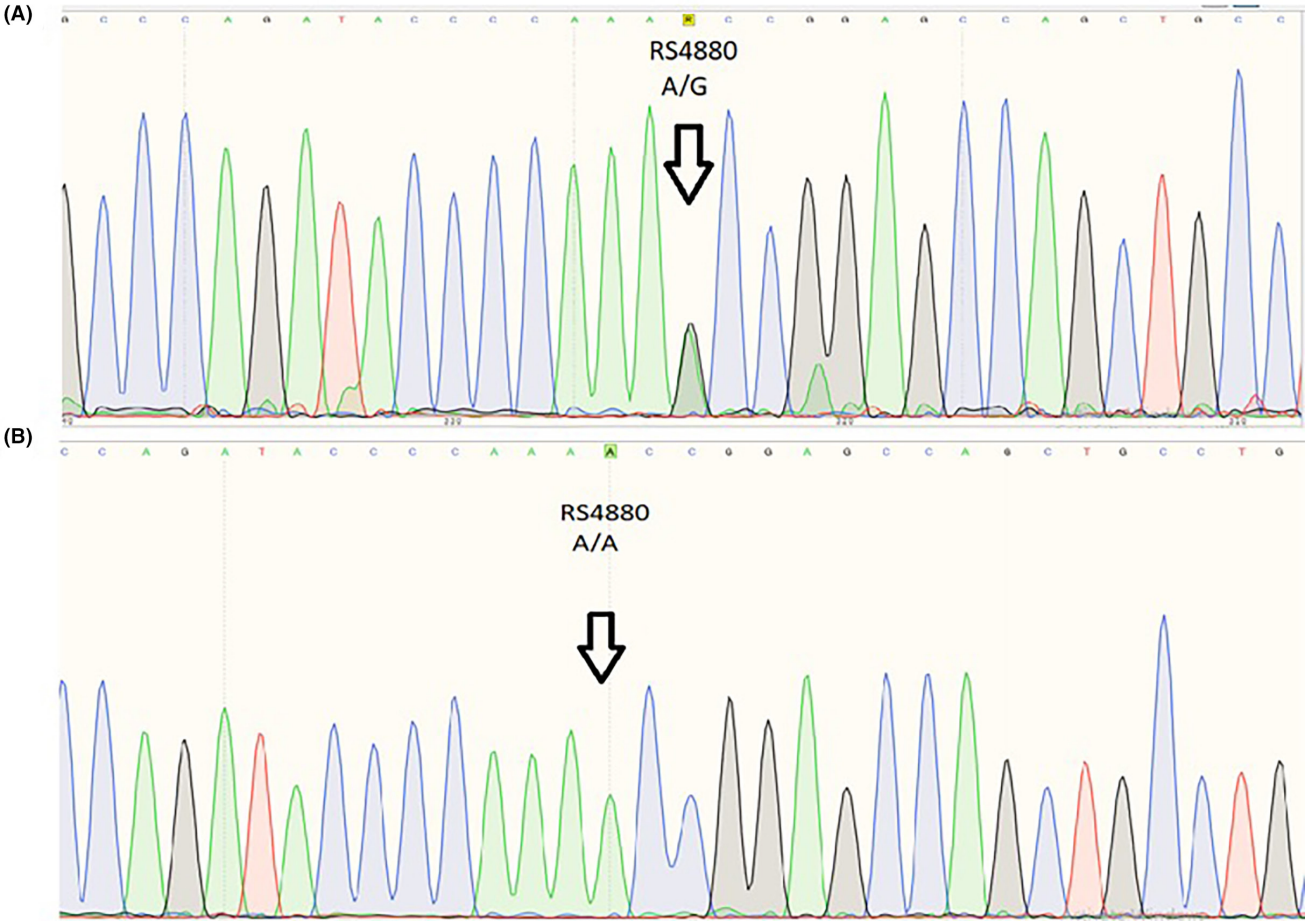
Sanger sequencing result of Type 2 diabetes mellitus (T2D) patient with rs4880 A.G. Heterozygous single nucleotide polymorphism as indicated by the arrow.

To validate the results, we used Sanger sequencing. Samples from patients with T2D without DN harboured the *SOD2* rs4880 A.G heterozygous SNP, and those from the control group were positive for *SOD2* rs4880 A.A homozygous SNP, as shown in Figures 3A,B.

## 4 DISCUSSION

T2D is a metabolic disorder characterized by hyperglycaemia and is associated with several complications, including DN, the leading cause of end‐stage renal disease worldwide. Recent studies have revealed an association between SNPs of antioxidant enzymes and improper defence against ROS, leading to intracellular accumulation of free radicals and subsequent oxidative stress.^24^ Many studies have suggested that oxidative stress is implicated in the development of T2D complications.^25^, ^26^, ^27^ Increasing free radicals and improper defence by antioxidants, can cause oxidative damage to lipids, proteins and nucleic acids.^28^ Genes encoding antioxidant enzymes show varying expression patterns across different populations, with some studies revealing weak or insignificant associations. However, other studies have indicated a significant association in certain populations, along with an increased risk of DN.^24^

SOD2 is located in the mitochondrial matrix and functions to convert superoxide free radicals into hydrogen peroxide.^29^ Polymorphisms involving the substitution of valine with alanine at position 16 in *SOD2* have been shown to decrease the formation of active SOD2 in the inner membrane of the mitochondrial matrix of the rat liver.^30^ Another study showed that the alanine variant increased the activity of SOD2 and its ability to neutralize the superoxide free radicals in leukocytes, thereby decreasing oxidized low density lipoprotein and the associated risk of coronary artery disease and myocardial infraction.^31^ However, under conditions of metabolic disruption such as that in T2D, the increase in SOD2 activity can lead to increased hydrogen peroxide accumulation, contributing to oxidative stress and insulin resistance.^32^

In this study, we investigated the association between SNPs of the antioxidant enzymes and T2D with or without DN. Our results showed a significant association between *SOD2* rs4880 and DN, but not with T2D. Notably, *SOD2* rs4880 was linked to a nearly threefold higher risk of DN in patients with DM than in healthy controls (OR = 2.99 [1.31–6.83]).

The results of a previous study conducted on the same population contradicts our results regarding *SOD2* rs4880 expression.^33^ However, our study included a healthy control group, leading to differences in inclusion criteria. Furthermore, we validated our results using robust Sanger sequencing. Interestingly, our results align with those of previous studies conducted on Japanese, Korean, Mexican, Finnish, Swedish, and Danish populations, where *SOD2* rs4880 was significantly associated with DN in patients with T2D.^14^, ^30^, ^34^, ^35^ Yahya et al. (2019) found that the *SOD2* rs4880 polymorphism was associated with development of DN in Malaysian patients with T2D,^36^ which aligns with our findings. Another study demonstrated a correlation between *SOD2* allelic variants and the onset and progression of DN, indicated by decreased eGFR, elevated plasma advanced oxidation protein products (AOPP) concentration, and reduced SOD2 activity in patients with type 1 diabetes (T1D).^37^ Collectively, these results indicate that patients with DM with this polymorphism are prone to increased levels of ROS, as reported previously,^35^, ^38^ reinforcing the role of oxidative stress in DN among patients with T2D.

In the present study, no association was found between *CAT* rs769217 and T2D or DN. This finding is similar to that of a previous study that reported no association between *CAT* rs769217 and acute kidney injury and idiopathic nephrotic syndrome in a Chinese Paediatric Population.^39^ Similarly, a study involving Caucasian‐Brazilian participants found no correlation between the *‐262C/T* polymorphism in the CAT gene and DN.^40^ However, another study conducted on the Hungarian population to examine the effects of the *CAT* rs769217 polymorphism on CAT activity found that the activity of the CAT enzyme was significantly decreased in cases of gestational diabetes and T2D.^41^ Kidir et al. (2016) also found an association between *CAT* rs769217 and acute kidney injury.^42^ *CAT* rs769217 has been shown to be associated with hospital morbidity and mortality in the Turkish population with acute kidney injury.^42^ In addition, Mohammedi (2013) found that the A allele of rs7947841 in the *CAT* gene was associated with an increased risk of DN in patients with T1D.^43^ Chistiakov (2006) found an association between the *‐262 T > C* polymorphism of the *CAT* gene and DN in Russian patients with T1D.^44^ These discrepancies in findings may be attributed to the ethnic characteristics or exogenous factors contributing to the genotype.

The primary limitation of this study is the small sample size; a larger sample size is needed to confirm our findings. Nevertheless, our study revealed significant differences between healthy controls and patients with T2D with DN regarding the expression of *SOD2* rs4880, which encodes the SOD2 antioxidant enzyme. *SOD2* rs4880 might increase the genetic susceptibility to DN and may therefore serve as a predictive marker. Our findings may aid in the design of antioxidant therapy to overcome oxidative stress and prevent the development and progression of DN in patients with T2D.

## AUTHOR CONTRIBUTIONS

Samar Sultan conceived and designed the study, Meshari Alharbi, Nuha Alrayes, Nehad Makki, Hanan Faruqui, Lama Basuni, Amani Alhozali, Reham Abdulnoor, and Mazin Almaghrabi collection of samples, Samar Sultan and Meshari Alharbi performed the data analyses, Samar Sultan and Meshari Alharbi interpretation of data and wrote the manuscript. Samar Sultan and Meshari Alharbi helped in the design of the study, data collection, and analyses. All of the authors read and approved the final manuscript.

## FUNDING INFORMATION

This research work was supported by institutional funds projects from the Ministry of education and King Abdulaziz University, Jeddah, Saudi Arabia under grant no (IFRC‐126‐290‐2020).

## CONFLICT OF INTEREST STATEMENT

The authors declare that there is no conflict of interest that could be perceived as prejudicing the impartiality of the research reported.

## ETHICS STATEMENT

The authors are accountable for all aspects of the work in ensuring that questions related to the accuracy or integrity of any part of the work are appropriately investigated and resolved. The study was approved by the Ethics Committee of the National Guard Hospital, Jeddah, Saudi Arabia (IRB#SP21J/419/09).

## Data Availability

Data sharing is not applicable to this article as no new data were created or analyzed in this study.N/A
